# Gray-Scale vs. Color Doppler Ultrasound in Cold Thyroid Nodules

**DOI:** 10.5539/gjhs.v7n3p147

**Published:** 2014-11-25

**Authors:** Mohammadgharib Salehi, Farhad Nalaini, Babak Izadi, Khosro Setayeshi, Mansour Rezaei, Seyyed Nooredin Naseri

**Affiliations:** 1 Department of Radiology, Imam Reza Hospital, Kermanshah University of Medical Sciences, Kermanshah, Iran; 2 Department of Pathology, Kermanshah University of Medical Sciences, Kermanshah, Iran; 3 Department of Surgery, Imam Reza Hospital, Kermanshah University of Medical Sciences, Kermanshah, Iran; 4 Biostatistics and Epidemiology Department, Social Development and Health Promotion Research Center, Kermanshah University of Medical Sciences, Kermanshah, Iran

**Keywords:** thyroid, nodule, gray-scale sonography, color Doppler sonography, malignant, benign

## Abstract

We intended to compare gray-scale vs. color Doppler ultrasound findings in cold thyroid nodules. Sixty-four patients with cold thyroid nodules for whom the presumptive diagnosis of malignancy (based on isotope scan study) had been made were consecutively included. They underwent gray-scale and color Doppler sonography studies. Based on histopathologic examination of surgically removed nodules, there were respectively 25 (39%) and 39 (61%) malignant and benign nodules. On color Doppler sonography, preference central hypervascularity was the most common finding in malignant nodules (17 nodules, 68%). Among benign nodules, preference perinodular hypervascularity was the most common finding (26 nodules, 66.7%). The most sensitive and specific Doppler sonography findings for malignant nodules were preference central hypervascularity (68%) and only central vascularity (97%), respectively. On gray-scale sonography, absent halo sign was the most common finding in malignant nodules (20 nodules, 80%). Among benign nodules, microcalcification was the most common finding which was reported in 12 nodules (30.7%). Hypoechogenicity was the most specific finding (76.9%) for malignant nodules. Since both gray-scale and color Doppler ultrasonography are inexpensive, non-invasive, and accessible methods to diagnose thyroid malignant cold nodules, it is recommended that these methods be applied by clinicians to assist or even substitute other invasive methods.

## 1. Introduction

Thyroid nodule is relatively common in general population. With introduction of medical imaging tools, the detection rate of thyroid nodules has increased (Manso García & Velasco Marcos, 2014; Bomeli, LeBeau, & Ferris, 2010). It is estimated that thyroid nodules can be as prevalent as 67% in general population (Singer, 1996). By keeping in mind this figure, healthcare-related costs and difficulties in their management arise as the high prevalence of thyroid nodules becomes more prominent. So radiologic images will help so much in clinical practice to deal with these nodules and help arrange further treatments. Among various radiologic methods, ultrasonography is by far the most widely used method to study thyroid nodules (Ezzat, Sarti, Cain, & Braunstein, 1994).

Overall, thyroid cancer is rare in humans (about 1% of all cancers) and mostly is of papillary carcinoma which is associated with good prognosis (Haber, 2000). Usually assessments done for evaluation of a suspicious thyroid nodule is costly and time-consuming. Fine needle aspiration is a standard way to yield beneficial information about the nature of the nodule. Besides that, radiologic images also provide useful information about thyroid nodules. There are advantages for radiologic examinations like being rapid and non-invasive. However, there is no way with 100% sensitivity or specificity that clinicians can rely on before surgical removal of the nodule. Sonography can identify nodules as small as 2 mm in diameter. Therefore, it is a valuable method in providing useful information about structure and anatomy of thyroid nodules and assists in doing other invasive techniques such as aspiration or surgery.

Previous studies have tried to find characteristics of nodules on sonography to help differentiate malignant from benign nodules (Algin et al., 2010; Slapa, Jakubowski, Slowinska-Srzednicka, & Szopinski, 2011; Popowicz, Klencki, Lewiński, & Słowińska-Klencka, 2009). Color Doppler sonography has been used previously for defining characteristics of malignant thyroid nodules (9). Gray-scale sonography is another method to describe thyroid nodules (Ma et al., 2014; Phuttharak, Somboonporn, & Hongdomnern, 2009; Varverakis, Neonakis, Tzardi, & Chrysos, 2007; Moon et al., 2012). All these efforts have been done owing to the fact that thyroid cancers grow slowly and if diagnosed at early stages, can be cured. Undoubtedly non-invasive accessible methods which can provide necessary information about the potential malignancy of a thyroid nodule will be regarded as valuable methods in clinical practice.

Here, we intended to compare the findings of gray-scale sonography and color Doppler ultrasound in cold thyroid nodules.

## 2. Patients and Methods

In this cross-sectional study, 64 patients with cold thyroid nodule for whom the presumptive diagnosis of malignancy (based on isotope scan study) had been made were consecutively included. They had been hospitalized in the surgery department of our university hospital and were candidate to undergo thyroid surgery to remove the nodule.

They underwent gray-scale and color Doppler sonography studies. The imaging variables documented were calcification, halo sign, and parenchymal echogenicity (gray-scale sonography) and resistance index (RI), vascularity and number of nodules (color Doppler sonography). All patients underwent surgical removal of the nodules. Categorization of benign vs. malignant nodules was based on histopathologic report of the surgically removed nodules.

The data gathered were entered into the SPSS software for Windows (ver. 20.0). Descriptive indices such as frequency, percentage, mean and its standard deviation (SD) were used to express the data. To compare the findings between two methods of sonography, the McNemar’s test was used and for correlation of the findings, kappa coefficient was calculated.

The study details were described for the patients and informed consent was obtained prior to sonography examination. The Ethics Committee of our medical school approved the protocol of this study.

## 3. Results

**Figure 1:**
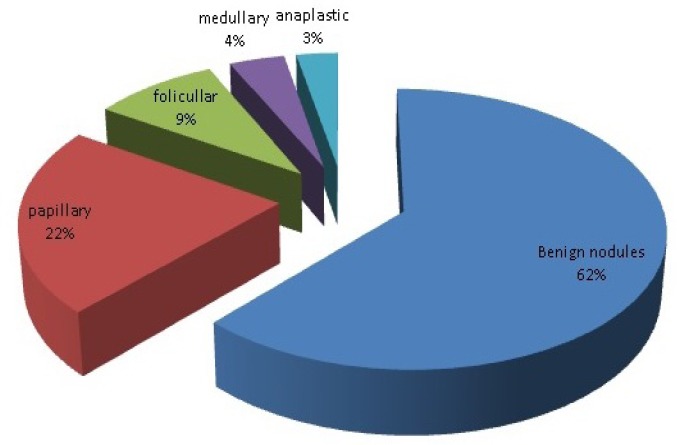
Distribution of 64 surgically removed benign and malignant thyroid nodules based on histopathologic examination

Mean (±SD) age of the patients was 42.4 (±11.9) years (range, 20-68 years). There were 46 women and 18 men. Based on histopathologic examination, there were 25 malignant nodules (39%, 17 females and 8 males) and 39 nodules were benign (61%, 29 females and 10 males). Frequency distribution of benign and malignant thyroid nodules is presented in Figure 1.

**Figure 2:**
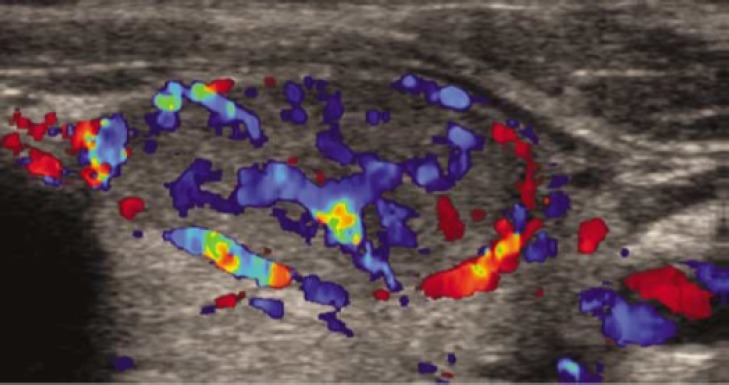
Preference central hypervascularity on color Doppler sonography as the most common finding in malignant nodules. There is marked intranodular blood flow and less significant perinodular blood flow

**Figure 3:**
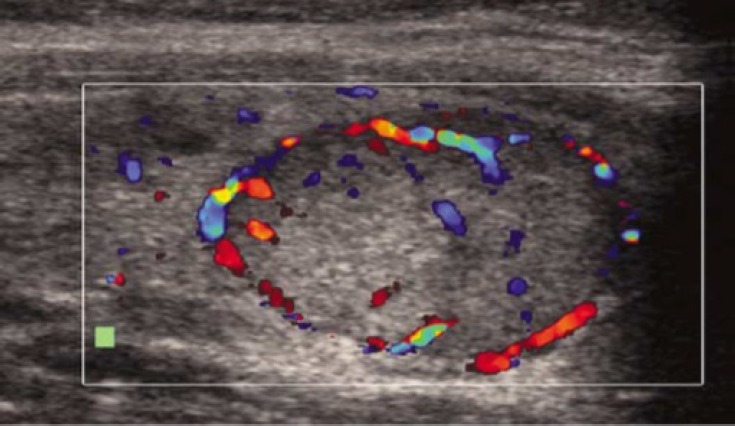
Preference perinodular hypervascularity with exclusively perinodular blood flow on color Doppler sonography as the most common finding in benign nodules

Table 1 presents color Doppler sonography findings in malignant and benign nodules. As shown, preference central hypervascularity was the most common finding on color Doppler sonography in malignant nodules which was found in 17 nodules (68%) (Figure 2). Among benign nodules, preference perinodular hypervascularity was the most common finding which was reported in 26 nodules (66.7%) (Figure 3). Only central vascularity was the most specific finding (97%) and preference central hypervascularity was the most sensitive finding (68%) for malignant nodules.

**Table 1:** Distribution of color Doppler sonography findings in malignant and benign cold thyroid nodules and related sensitivity and specificity values of each finding for malignant nodule

	Malignant nodules (N= 25)	Benign nodules (N= 39)	Sensitivity	Specificity
Absent vascularity	3 (12%)	9 (23.1%)	3/25= 12%	30/39= 76.9%
Preference perinodular hypervascularity	4 (16%)	26 (66.7%)	4/25= 16%	13/39= 33.3%
Preference central hypervascularity	17 (68%)	4 (10.2%)	17/25= 68%	35/39= 89.7%
Only central vascularity	1 (4%)	1 (2.5%)	1/25= 4%	38/39= 97%

**Figure 4:**
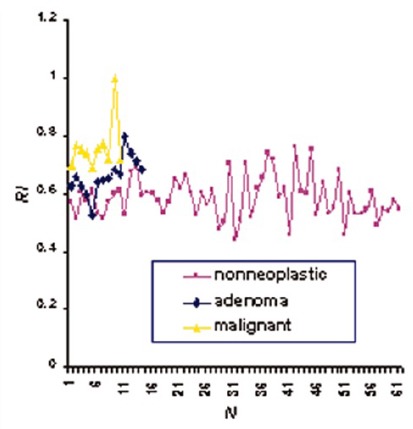
Graphic representation of a nodule’s type based on the associated resistant index (RI) on color Doppler sonography.

Table 2 presents gray-scale sonography findings in malignant and benign nodules. As shown, absent halo sign was the most common finding on gray-scale sonography in malignant nodules which was found in 20 nodules (80%). Among benign nodules, microcalcification was the most common finding (12 nodules, 30.7%). Hypoechogenicity was the most specific finding (76.9%). Figure 4 shows resistant index based on color Doppler sonography findings.

**Table 2:** Distribution of gray-scale sonography findings in malignant and benign cold thyroid nodules and related sensitivity and specificity values of each finding for malignant nodule

	Malignant nodules (N= 25)	Benign nodules (N= 39)	Sensitivity	Specificity
Absent halo sign	20 (80%)	11 (28.2%)	20/25= 80%	28/39= 71.7%
Microcalcification	18 (72%)	12 (30.7%)	18/25= 72%	27/39= 69.2%
Hypoechogenicity	14 (56%)	9 (23%)	14/25= 56%	30/39= 76.9%

## 4. Discussion

The findings we observed here indicate that the imaging findings of absent halo sign, hypoechogenicity, and microcalcifications on gray-scale sonography were more likely indicative of malignancy in thyroid cold nodules. On the other hand, only central vascularity, preference central hypervascularity, and higher RI on color Doppler ultrasonography were more likely to be associated with malignant thyroid cold nodules.

Thyroid nodules are commonly encountered in clinical practice. Since most thyroid malignant nodules can be easily treated, prompt and early diagnosis is of crucial importance. According to our findings both gray-scale and color Doppler ultrasonography methods were useful in detection of malignant nodules. These findings are compatible with former studies. Sonographic features which have been reported as having predictive value for malignancy include microcalcifications of less than 2 mm, marked hypoechogenicity, irregular margins, solid composition, absence of a hypoechoic halo around the nodule, size >1 cm, taller-than-wide-shape, and an intra-nodular vascularity (Hoang, Lee, Lee, Johnson, & Farrell, 2007; Chaudhary & Bano, 2013). In another study (Ginat, Butani, Giampoli, Patel, & Dogra, 2010), the authors reviewed ultrasound appearances of thyroid nodules with regard to morphological features of 156 thyroid nodules. They reported that microcalcifications, coarse internal calcifications, markedly hypoechoic components, mostly solid-to-solid contents, infiltrative or microlobulated margins, and taller-than-wide shape were findings which were more indicative of malignant nodules. On the other hand, peripheral calcification and purely cystic composition were most likely to be associated with benign nodules. Ma et al. (2014) examined 172 nodules in 144 patients to find any association between gray-scale, contrast-enhanced ultrasonography, and color Doppler ultrasonography methods with histopathologic examination of the nodules. There were 78 benign and 94 malignant nodules. They found that ring enhancement and homogeneity of enhancement on contrast-enhanced ultrasonography and microcalcification and halo sign on gray-scale ultrasonography were the most useful indicators of malignancy with a high odds ratio. Gray-scale USG features of thyroid nodules are useful to distinguish patients with clinically significant thyroid nodules from those with innocuous nodules despite the overlap of findings. From our study, it is apparent that the USG findings of poorly defined margins, marked Hypoechogenicity, microcalcifications, and a taller-than-wider shape had also been demonstrated as being associated with a high diagnostic accuracy for identifying malignant thyroid nodules (Popli, Rastogi, Bhalla, & Solanki, 2012).

However, some authors believe that even though ultrasound yields valuable information about characteristics of malignant or potentially malignant thyroid nodules, no single ultrasound finding can be completely referred to as able to predict with complete assurance the prediction of malignant lesions and fine needle aspiration is inevitable in pre-operative work-up (Yunus & Ahmed, 2010).

## 5. Conclusion

Since both gray-scale and color Doppler ultrasonography are inexpensive, non-invasive, and accessible imaging tools to diagnose malignant thyroid cold nodules, it is recommended that these methods be applied more by clinicians to assist or substitute other invasive methods.
